# A case of benign immunoglobulin D monoclonal gammopathy of undetermined significance with 26 years of follow‐up

**DOI:** 10.1002/jha2.846

**Published:** 2024-01-10

**Authors:** Larry D. Anderson, Joan Bladé, Robert A. Kyle

**Affiliations:** ^1^ Myeloma, Waldenstrom's, and Amyloidosis Program Hematologic Malignancies and Cellular Therapy Program Simmons Comprehensive Cancer Center UT Southwestern Medical Center Dallas Texas USA; ^2^ Department of Hematology Amyloidosis and Myeloma Unit Institut d'Investigacions Biomèdiques August Pi i Sunyer Hospital Clínic de Barcelona University de Barcelona Barcelona Spain; ^3^ Division of Hematology Mayo Clinic Rochester Minnesota USA

**Keywords:** Immunoglobulin D (IgD), MGUS, monoclonal gammopathy

## Abstract

The presence of a serum immunoglobulin D (IgD) monoclonal protein (M‐protein) is seen in < 1% of patients with monoclonal gammopathies and is usually indicative of a malignant plasma cell disorder. Only a few cases of well‐documented benign monoclonal gammopathy of undetermined significance (MGUS) of IgD subtype have been reported, and only 2 of those had over 5 years of follow‐up at the time they were reported. Herein we describe longer‐term follow‐up of one of those 2 patients who has subsequently passed away from unrelated causes but never developed multiple myeloma or amyloidosis after 26 years of follow‐up. Although IgD MGUS is extremely rare, this case confirms that presence of an IgD M‐Protein is not always synonymous with a malignant plasma cell process.

1

The presence of a serum immunoglobulin D (IgD) monoclonal protein (M‐protein) is seen in < 1% of patients with monoclonal gammopathies and is usually indicative of a malignant plasma cell disorder [1, 2, 3, 4]. Only a few cases of well‐documented benign monoclonal gammopathy of undetermined significance (MGUS) of IgD subtype have been reported, and only 2 of those had over 5 years of follow‐up at the time they were reported [5, 6, 7]. Herein we describe longer‐term follow‐up of one of those 2 patients who has subsequently passed away from unrelated causes but never developed multiple myeloma (MM) or amyloidosis after 26 years of follow‐up [5]. Although IgD MGUS is extremely rare, this case confirms that presence of an IgD M‐Protein is not always synonymous with a malignant plasma cell process.

MGUS is characterized by the presence of a serum M‐protein of < 3.0 grams per deciliter (g/dL), clonal plasma cells < 10% in the bone marrow, and the absence of CRAB features (hypercalcemia, renal insufficiency, anemia, or bone lesions attributable to clonal plasma cells) [8, 9]. MGUS is seen in 3.2% of people ≥ 50 years of age and 5.3% of those ≥ 70 years of age, and about one‐third of these develop MM over their lifetime at a rate of around 1% per year [10]. It has also been observed that MGUS precedes the diagnosis of MM in most cases [11]. The risk of progression increases with M‐protein levels over 1.5 g/dL, elevated serum free light chains, or non‐IgG heavy chain subtype [10]. Most patients with an M‐protein from plasma cell disorders have an IgG M‐protein subtype (70%–81%), and less commonly an IgA (12%–13%) or IgM (2%–16%), but only rarely (< 1%) do patients present with an IgD or IgE M‐protein [10, 12]. Patients with an IgD M‐protein often have poor prognosis due to either aggressive myeloma or amyloidosis despite a smaller amount of M‐protein that is often unquantifiable, partly due to shorter half‐life of IgD compared to IgG and less secretion of IgD by the plasma cells [13, 14].

We now report an update on our case of IgD MGUS which was originally published in 1994 [5]. The case was previously reported after 8 years of follow‐up from initial diagnosis. The diagnosis was originally made in June 1985 after the incidental finding of an M‐protein on serum protein electrophoresis (SPEP) as part of her evaluation for Parkinson's disease at age 59. After 10 years she was lost to follow‐up at the Mayo Clinic until she eventually presented for follow‐up at UT Southwestern Medical Center in Dallas, Texas in 2009 to establish care after moving to be with family. On presentation in August 2009, her laboratory workup showed features initially worrisome for disease progression with creatinine rising to 2.15 mg/dL and hemoglobin dropping to 8.3 g/dL and normal calcium. However, a thorough workup in 2009, fortunately, confirmed no signs of progression to myeloma or amyloidosis, and her anemia and acute kidney injury issues were attributable to GI blood loss, hypotension related to antihypertensives and dehydration with subsequent improvement of her creatinine to 1.3 mg/dL a month after stopping one antihypertensive and reducing the dose for the other one. Her creatinine later improved to normal 0.60 mg/dL within the next 6 months, along with an improvement of hemoglobin to 10.9 g/dL. Bone marrow biopsy showed 1.2% lambda‐restricted aberrant plasma cells on flow cytometry and 5% plasma cells on bone marrow morphologic differential with a negative stain for Congo red (Figure 1). Fat pad biopsy was also negative for amyloidosis. Serum total IgD level was 1370 mg/L in 1985, 1002 mg/L in 1993, and only slightly higher at 2280 mg/L in 2009. SPEP/immunofixation continued to show the presence of an unquantifiable but detectable IgD lambda M‐protein in 2009 (from 0.5 g/dL in 1985, 0.85 g/dL in 1989, and 1.1 g/dL in the beta region in 1993), and lambda free light chains were elevated at 89 mg/L with kappa/lambda ratio suppressed at 0.0137. Twenty‐four‐hour urine total protein had minimally increased from 100 mg/day in 1985, 125 mg per day in 1989 (with lambda light chain M‐protein 50 mg/day), and 166 mg/day in 1993 to 196 mg/day in 2009 (at the time of unrelated acute kidney injury, along with M‐protein 153 mg/day). Cardiac troponin was mildly elevated during the episode of acute kidney injury but then normalized again. Skeletal survey radiographs showed only degenerative changes with no lytic bone lesions.

**FIGURE 1:**
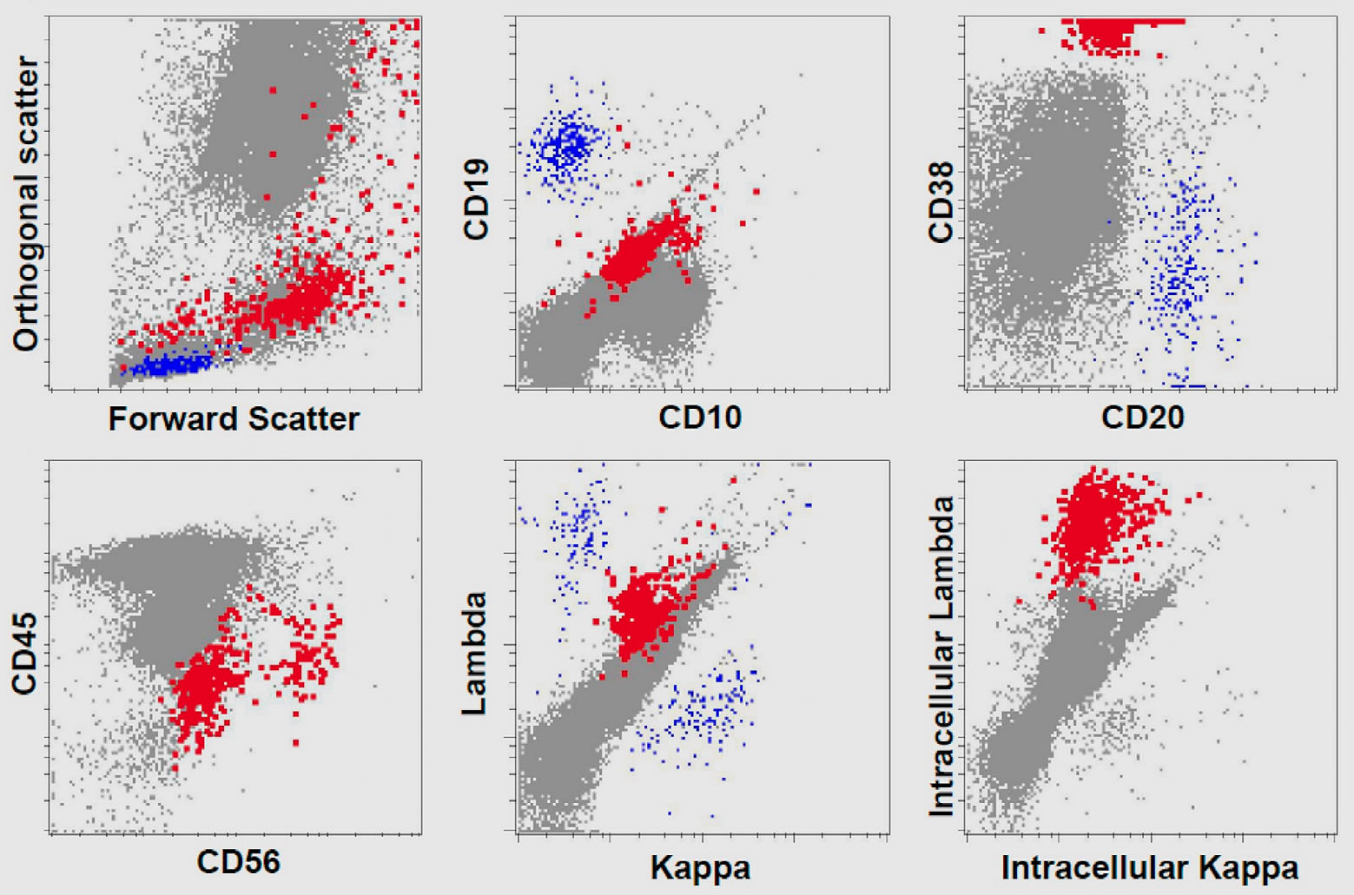
Bone marrow flow cytometry. Immunophenotypic analysis of bone marrow reveals a 1.2% population of variably‐sized plasma cells with the following immunophenotype: CD10(‐), CD19(predominantly ‐), CD20(‐), CD38(bright +), CD45(dim +), CD56(subset +), surface kappa(‐), surface lambda(partial dim +), intracellular kappa(‐), intracellular lambda(+).

Although she had no issues related to her M‐protein, her Parkinson's continued to progress, and she required a walker and assistance with activities of daily living by 2009. Ultimately during a hospital admission for gastrointestinal (GI) bleeding in January 2010, colonoscopy showed pseudomembranous colitis from *clostridium difficile* (with no signs of amyloidosis on biopsy as a cause for her GI bleed), and she ended up having a large cerebrovascular accident (large right cerebral hemisphere infarct and left temporal lobe infarct) that led to discharge to hospice care in January 2010 at her family's request. She ultimately passed away at the age of 85 in June 2011 while in hospice. Even though a decade has passed since her death, we feel that this long‐term follow‐up will still be important to report since to this date no additional new cases of IgD MGUS with long‐term follow‐up > 5 years have been published to our knowledge.

## AUTHOR CONTRIBUTIONS

Larry D Anderson Jr designed and wrote the first draft of the paper, interpreted the results, organized the figures, and compiled the reference list. Blade and Kyle published the initial report as well as interpreted the results and edited the follow‐up paper. All authors were involved in the revision of the manuscript and approved the final version.

## CONFLICT OF INTEREST STATEMENT

Larry D. Anderson Jr. reports honoraria from consulting and scientific advisory board activity from BMS, Celgene, Janssen, Amgen, GSK, Karyopharm, and AbbVie. He serves on a DSMB for Prothena and has received institutional research funding for clinical trials from BMS, Celgene, Janssen, GSK, and AbbVie.

## FUNDING INFORMATION

The authors did not receive funding from any organization for the submitted work.

## ETHICS STATEMENT

The authors have confirmed ethical approval statement is not needed for this submission.

## PATIENT CONSENT STATEMENT

The authors have confirmed patient consent statement is not needed for this submission.

## Data Availability

The data that support the findings of this study are available upon reasonable request from the corresponding author.
